# Effect of early determinants on adolescent fat-free mass: RPS cohort of São Luís – MA

**DOI:** 10.11606/s1518-8787.2020054002229

**Published:** 2020-11-12

**Authors:** Raina Jansen Cutrim Propp Lima, Rosângela Fernandes Lucena Batista, Cecília Claudia Costa Ribeiro, Vanda Maria Ferreira Simões, Pedro Martins Lima, Heloisa Bettiol, Antônio Augusto Moura da Silva

**Affiliations:** I Instituto Federal de Educação Ciência e Tecnologia do Maranhão Departamento de Ensino AçailândiaMA Brasil Instituto Federal de Educação. Ciência e Tecnologia do Maranhão. Departamento de Ensino. Açailândia, MA, Brasil; II Universidade Federal do Maranhão Centro de Ciências Biológicas e da Saúde Departamento de Saúde Pública São LuísMA Brasil Universidade Federal do Maranhão. Centro de Ciências Biológicas e da Saúde. Departamento de Saúde Pública. São Luís, MA, Brasil; III Universidade Federal do Maranhão Centro de Ciências Biológicas e da Saúde Departamento de Odontologia II São LuísMA Brasil Universidade Federal do Maranhão. Centro de Ciências Biológicas e da Saúde. Departamento de Odontologia II. São Luís, MA, Brasil; IV Universidade Federal do Maranhão Centro de Ciências Sociais, Saúde e Tecnologia ImperatrizMA Brasil Universidade Federal do Maranhão. Centro de Ciências Sociais, Saúde e Tecnologia. Imperatriz, MA, Brasil; V Universidade de São Paulo Faculdade de Medicina de Ribeirão Preto Departamento de Puericultura e Pediatria Ribeirão PretoSP Brasil Universidade de São Paulo. Faculdade de Medicina de Ribeirão Preto. Departamento de Puericultura e Pediatria. Ribeirão Preto, SP, Brasil

**Keywords:** Adolescent Health, Fetal Development, Body Composition, Biological Factors, Social Determinants of Health, Socioeconomic Factors

## Abstract

**OBJECTIVE:**

To analyze the effects of early determinants on adolescent fat-free mass.

**METHODS:**

A cohort study with 579 adolescents evaluated at birth and adolescence in a birth cohort in São Luís, Maranhão. In the proposed model, estimated by structural equation modeling, socioeconomic status (SES) at birth, maternal age, pregestational body mass index (BMI), gestational smoking, gestational weight gain, type of delivery, gestational age, sex of the newborn, length and weight at birth, adolescent socioeconomic status, “neither study/nor work” generation, adolescent physical activity level and alcohol consumption were tested as early determinants of adolescent fat-free mass (FFM).

**RESULTS:**

A higher pregestational BMI resulted in higher FFM in adolescence (Standardized Coefficient, SC = 0.152; p < 0.001). Being female implied a lower FFM in adolescence (SC = −0.633; p < 0.001). The negative effect of gender on FFM was direct (SC = −0.523; p < 0.001), but there was an indirect negative effect via physical activity level (SC = −0.085; p < 0.001). Women were less active (p < 0.001). An increase of 0.5 kg (1 Standard Deviation, SD) in birth weight led to a gain of 0.25 kg/m^2^ (0.106 SD) in adolescent FFM index (p = 0.034). Not studying or working had a negative effect on the adolescent's FFM (SC = −0.106; p = 0.015). Elevation of 1 SD in the adolescent's physical activity level represented an increase of 0.5 kg/m^2^ (0.207 SD) in FFM index (p < 0.001).

**CONCLUSIONS:**

The early determinants with the greatest effects on adolescent FFM are gender, adolescent physical activity level, pregestational BMI, birth weight and belonging to the “neither-nor” generation.

## INTRODUCTION

According to the developmental origin of health and disease theory, events occurring during early developmental phases starting from fetal life may be related to determined lifelong health and disease patterns ^1^ . The prenatal period may influence the distribution of body composition components throughout life, also being affected by maturation and aging, as well as other factors such as diseases and behavioral characteristics ^2^^,^^3^ .

Many studies have focused on early determinants as predictors of future body fat, but few have verified the effect of these determinants on fat-free mass (FFM) during different stages of life ^3^ . Fat-free mass is a clinical indicator of health and a determinant of functional capacity, being associated with a longer survival of patients with heart disease and cancer, among others ^4^^–^^6^ . Its conservation is important for survival during aging ^7^ . Contemporary aspects related to lifestyle such as physical activity and eating habits have been well established as factors influencing the FFM of an individual ^8^^–^^10^ . However, earlier aspects such as growth during gestation, childhood and adolescence have been investigated, although the available scientific literature is scarce and controversial ^3^^,^^11^ .

The major early determinants of FFM at the beginning of adult age are weight and length at birth, as well as maternal (height, socioeconomic conditions and educational level) and gestational (gestational age, pre-gestational weight, smoking during pregnancy) variables ^3^ . A low birth weight (BW) is associated with a smaller FFM during adulthood, contributing to the risk of sarcopenia and functional disability at the end of life ^12^ , whereas a greater BW may be associated with a greater FFM (especially in men) at 60-64 years ^11^ . However, it is still unknown whether the effects previously observed on body composition reflect a totally intrauterine programming. There may be confusion due to lifestyle or genetics, in addition to possible pathways mediated by other exposures, which would represent a problem ^13^ .

Also, most of the available evidence has used body mass index (BMI) to assess nutritional status although studies using this tool cannot elucidate whether the associations reflect the influence of growth on fat mass (FM), on FFM, or on both ^11^ . Furthermore, a systematic review observed that the studies use linear regression for statistical analysis ^3^ . There is criticism in the literature regarding linear regression since it only investigates direct relationships between the explanatory variables and the outcome, without evaluating the effects of indirect pathways through the mediating variables ^14^^,^^15^ .

Understanding the effects of early determinants of future FFM and how body composition behaves over the years using methods that can detect differences between body composition compartments regarding a birth cohort and appropriate statistical analysis is of fundamental importance for the elaboration of public health strategies to promote health and prevent disorders related to reduced FFM.

Thus, the objective of this study was to answer the following questions: what are the major early determinants of FFM in adolescence? Do birth-related factors have a greater effect on FFM during adolescence than factors related to adolescence itself? Are all effects direct or they occur through mediating variables?

## METHODS

This was a cohort study based on individuals born in the city of São Luís, Maranhão, Brazil, involving three different periods. This cohort is included in the study “Life-long determinants of obesity, precursors of chronic disease, human capital, and mental health” conducted by the RPS consortium with the Universidade Federal do Maranhão (UFMA), Faculdade de Medicina de Ribeirão Preto (USP), and Universidade Federal de Pelotas (UFPel).

In São Luís, the subjects of this cohort were evaluated in three phases of life: at birth, in childhood (7 to 9 years) and currently in adolescence (18/19 years). Our study used data collected in the first and third phases of the cohort in the city of São Luís. Initially, the study was conducted in ten public and private hospitals from March 1997 to February 1998. The study sample base included 96.3% of births, excluding non-hospital births and births that occurred in hospitals where fewer than 100 deliveries occurred per year. One in seven births at each maternity hospital was recruited for the study, with proportional sharing of the number of births in each unit. The interviews were conducted at the hospital or at home, and data were collected using the Birth and Interview Recording Card, the Standardized Questionnaire, and the Mortality Investigation Card ^16^ . We included 2,541 births, with 5.8% loss due to refusal or early discharge.

All subjects included in the first phase were located in the four Military Enlistment branches of São Luís, in the school census of 2014 and in universities. Printed and virtual advertising material, radio and TV networks and social media including WhatsApp and a site especially set up for this purpose were used for dissemination. The subjects identified (n = 684) were invited to attend follow-up. The evaluation focused on outcomes related to nutrition, body composition, precursor factors for chronic diseases, mental health, and human capital (schooling, income and cognitive skills).

The study was conducted with 684 adolescents that were evaluated at birth and during adolescence. Exclusion criteria were twins, subjects who were not born in São Luís and subjects who had no FFM or height data, which were necessary for the construction of the outcome variable. Thus, the final sample consisted of 579 adolescents of both sexes (18/19 years).

Data from the third phase were collected by properly trained students and health graduates. A pilot study was carried out with simulation of all stages of the research, for checking and technical adjustments. Questionnaires used in the study were validated but no reproducibility analysis was carried out. Data were collected in sequentially organized stations including different questionnaires (with questions about socioeconomic, personal and family data, health, physical activity, leisure, sedentary behavior and life habits) and evaluation instruments such as BodPod and densitometry (DEXA).

The DEXA station (Dual Energy X-Ray Absorptiometry) was equipped with a Lunar Prodigy GE Healthcare^®^ model for the measurement of body components and for the estimate of localized fat percent. This machine requires about 15 minutes for whole body scanning and 3 minutes for the examination of each bone density site. The participants were instructed to wear standard clothing, a swimming cap and to remove any accessories. They were previously weighed and measured and then positioned for the measurements of whole-body composition and of lumbar spine and femoral head bone mass. The instrument also provided the measurement of FM and FFM of each adolescent. Data collection and entry were performed using the Research Electronic Data Capture (RedCap).

In the theoretical model proposed, the variables of mother and newborn, as well as the variables of the adolescents, determine the FFM of the adolescent. The socioeconomic status at birth and during adolescence represents latent variables constructed from other variables observed. All the other variables were observed ( Figure 1 ). The socioeconomic status (SES) construct for both birth and adolescence was derived from the variables schooling of the family head (at birth and adolescence – never studied, 1 to 4 years, 5 to 8 years, 9 to 11 years, and 12 or more years of study) and monthly family income (birth and adolescence – minimum wages (MW); the national minimum wage was R$120.00 in 1997 and R$ 880.00 in 2016: ≤1; 1.1 to 1.9; 2 to 2.9; 3 to 4.9; 5 to 9.9, and ≥10).

**Figure:**
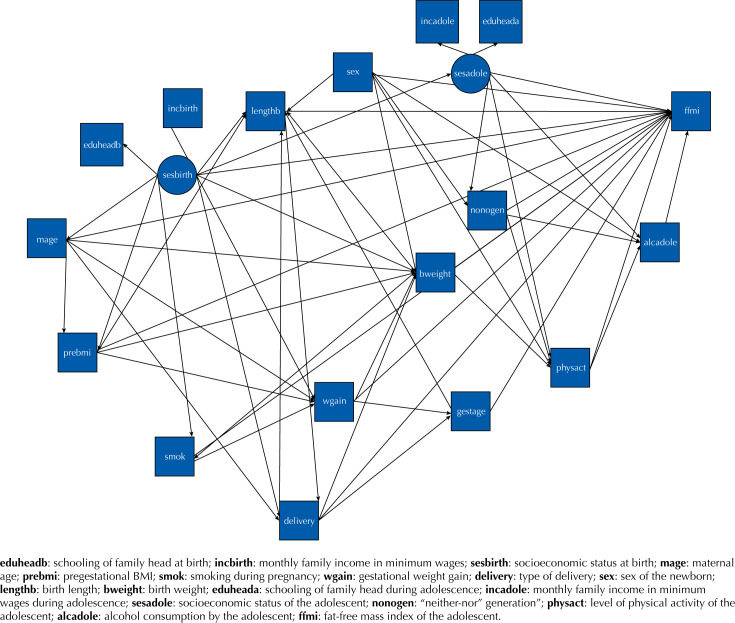
Theoretical model of the associations of observed and latent variables with the fat-free mass of adolescents of the RPS cohort of São Luís. São Luís, Brasil, 2016.

The main explanatory variables were birth weight and level of physical activity of the adolescent. Birth weight (kg) was recorded in a continuous manner in the model. The level of physical activity was determined using the 24-h Physical Activity recall survey elaborated from an adaptation of the Self-Administered Physical Activity Checklist – SAPAC ^17^ . The physical activity index (AI) used was the sum of all activities carried out during the week [time of activity per day × MET (metabolic equivalent of the task) × number of days of activity per week]. This variable was categorized as: sedentary (AI = 0), low (1 ≤ AI < 600), moderate (600 ≤ AI < 3000), and high (AI ≥ 3000).

The maternal variables analyzed were maternal age (years, treated as a continuous numerical variable), pregestational BMI (kg/m ^2^ , treated as a continuous numerical variable), smoking during pregnancy (answer to the question “Do you smoke?,” which may have been no or yes), gestational weight gain (kg, treated as a continuous numerical variable), type of delivery (vaginal or cesarean) and gestational age (weeks, treated as a continuous numerical variable).

The variables for the newborn were sex (male or female) and birth length (cm, treated as a continuous numerical variable). The following variables were also used for the adolescents: “neither-nor” generation (studies and/or works and does not study nor work), and alcohol consumption (measured with the Alcohol Use Disorder Identification Test (AUDIT) and categorized as low, risky, harmful or probable dependence) ^18^ .

The dependent variable was the quantity of FFM of the adolescents measured by DEXA and expressed as fat-free mass index (FFMI) (kg/m ^2^ ) and treated as a continuous numerical variable. This index is calculated by dividing adolescent FFM (kg) by height squared (m) ^19^ .

Structural equation modeling was used to investigate the effect of the explanatory variables and covariables on the FFM of the adolescent. This modeling has the advantage of dealing simultaneously with multiple dependence relations and is able to represent non-observed concepts (latent variables) in these relations by modeling the error of measurement in the estimation process ^20^ .

According to the theory proposed here, birth SES, maternal age, pregestational BMI, smoking during pregnancy, gestational weight gain, type of delivery, gestational age, sex of the newborn, birth length, BW, SES of the adolescent, “neither-nor” generation, level of physical activity, and alcohol consumption by the adolescent had a direct effect on the FFM of the adolescent.

Data were analyzed statistically using the Mplus software version 7. The Weighted Least Squares Mean and Variance Adjusted estimator (WLSWV) was used for the continuous and categorical variables. The THETA parameterization was used to control differences in residual variances. The following fit indices were considered in order to determine if the model showed good fit: a) p > 0.05 for the chi-square test (χ ^2^ ) ^14^ ; b) value < 0.05 and an upper limit of the 90% confidence interval of less than 0.08 for the root mean square error of approximation (RMSEA) ^15^ , and c) values of more than 0.95 for the Comparative Fit Index and the Tucker Lewis Index (CFI/TLI) ^15^ .

In the analysis of the standardized estimates for the construction of the latent variables, a factor loading of more than 0.5 with p < 0.05 indicated a correlation of moderately high magnitude between the variables observed and the construct ^14^ . In order to obtain suggestions of changes in the initial hypotheses we estimated modification indices using the *modindices* command, whereby the proposed modifications considered to be plausible from a theoretical viewpoint and with a value of the modification index higher than 10 could be incorporated, generating a new model ^15^ .

The total, direct and indirect effects of the latent and observed variables were evaluated in the final model. An effect was judged to be present when p < 0.05. The mean and standard deviation of the continuous variables was calculated in order to facilitate interpretation of the results. The result of the effect of the variable on the original metric was obtained by multiplying the value of the standardized coefficient of the total effect by the standard deviation of the variable (SC x SD).

To verify possible selection bias, the variables family income at birth and sex of the newborn were compared between those followed up and those lost to follow up. No formal sample size estimate was performed as it is not easily available in most used statistical programs, but we used as a rule of thumb a minimum of 20 observations per each variable included in the model. A sample size of 579 allowed us to include a maximum of 29 variables in the structural equation model, and we have included 17 variables.

This study complied with the formal requirements contained in the national and international standards of regulatory research involving human subjects.

## RESULTS

Of the 579 adolescents studied, 59.4% were males, 77% reported studying or working, and 37.1% were sedentary. Mean pregestational BMI was 21.1 (SD = 2.8) kg/m ^2^ and BW was 3.2 (SD = 0.5) kg. Mean adolescent FFMI was 16 (SD = 2.4) kg/m ^2^ . The remaining results are listed in Table 1 .

**Table 1:** Socioeconomic, demographic, family and adolescent characteristics of the RPS cohort of São Luís. São Luís, Brazil, 2016.

Variables		n	%
Sex of the newborn			
	Male	344	59.4
	Female	235	40.6
Head of family schooling at birth (years)			
	Never studied	5	0.9
	1 to 4	61	10.5
	5 to 8	168	29.0
	9 to 11	253	43.7
	≥ 12	28	4.8
	Unknown	64	11.1
Family income at birth (minimum wages)			
	≤1	77	13.3
	1.1 to 1,9	96	16.6
	2 to 2.9	95	16.4
	3 to 4,9	124	21.4
	5 to 9,9	97	16.8
	≥ 10	55	9.5
	Unknown	35	6.0
Smoking during pregnancy			
	No	549	94.8
	Yes	30	5.2
Type of delivery			
	Vaginal	355	61.3
	Cesarean	224	38.7
Head of family schooling at adolescence (years))			
	Never studied	11	1.9
	1 to 4	139	24.0
	5 to 8	288	49.7
	9 to 11	19	3.3
	≥ 12	60	10.4
	Unknown	62	10.7
Family income at adolescence (minimum wages)			
	≤ 1	114	19.7
	1.1 to 1.9	128	22.1
	2 to 2.9	118	20.4
	3 to 4.9	87	15.0
	5 to 9.9	52	9.0
	≥ 10	20	3.4
	Unknown	60	10.4
“Neither-nor generation”			
	Studies or works	446	77.0
	Does not study or work	133	23.0
Level of physical activity of the adolescent			
	Sedentary	215	37.1
	Low	81	14.0
	Moderate	136	23.5
	High	144	24.9
	Unknown	3	0.5
Alcohol consumption by the adolescent			
	Low	471	81.4
	Risky	90	15.5
	Harmful	10	1.7
	Probable dependence	8	1.4
	Total	579	100.0
		**Mean**	**Standard deviation**
Maternal age (years)		23.4	5.3
Pregestational BMI (kg/m ^2^ )		21.1	2.8
Gestational weight gain (kg)		9.7	5.0
Gestational age (weeks)		38.9	2.4
Birth weight (kg)		3.2	0.5
Birth length (cm)		48.9	2.4
Fat-free mass index of the adolescent (kg/m ^2^ )		16.0	2.4

The theoretical model showed good fit according to the RMSEA and CFI indicators, with no plausible suggestion of modification ( Table 2 ). The latent variables SES at birth and at adolescence had all indicators showing factor loadings higher than 0.5 (p < 0.001) ( Table 3 ).

**Table 2:** Fit indices of the model for the fat-free mass outcome of the adolescents in the RPS cohort of São Luís. São Luís, Brazil, 2016.

Indices		Model a
χ ^2^ b		
	Value	137.594
	Degrees of freedom	78
	p-value	< 0.001
RMSEA c		
	Value	0.036
	90% confidence interval	0.026–0.046
	p-value	0.990
CFI d		0.963
TLI e		0.936

a Initial and final model since there was no suggestion of plausible modification.

b Chi-square test.

c Root mean square error of approximation.

d Comparative Fit Index.

e Tucker Lewis Index.

**Table 3:** Standardized coefficients, standard errors and p-values of the direct effects of the observed variables and constructs on fat-free mass of adolescents of the RPS cohort of São Luís. São Luís, Brazil, 2016.

Pathways and estimates			Standardized coefficient	Standard error	p-value
Latent variables					
	SES at birth				
		Head of family schooling at birth	0.713	0.040	< 0.001
		Family income at birth	0.682	0.038	< 0.001
	SES of the adolescent				
		Head of family schooling during adolescence	0.649	0.050	< 0.001
		Family income during adolescence	0.526	0.046	< 0.001
Direct effects					
	Fat-free mass of the adolescent				
		SES at birth	0.194	0.060	0.578
		Maternal age	-0.130	0.040	0.001
		Pregestational BMI	0.146	0.036	< 0.001
		Smoking during pregnancy	0.048	0.058	0.403
		Gestational weight gain	0.041	0.042	0.338
		Type of delivery	0.081	0.056	0.151
		Gestational age	-0.025	0.036	0.478
		Sex of the newborn	-0.523	0.030	< 0.001
		Birth length	-0.043	0.051	0.399
		Birth weight	0.121	0.051	0.019
		SES of the adolescent	-0.282	0.350	0.421
		“Neither-nor” generation	-0.077	0.045	0.084
Level of physical activity of the adolescent			0.191	0.044	< 0.001
		Alcohol consumption by the adolescent	0.054	0.052	0.300
Maternal age					
		SES at birth	0.278	0.044	< 0.001
Pregestational BMI					
		SES at birth	-0.088	0.055	0.109
		Maternal age	0.299	0.043	< 0.001
Smoking during pregnancy					
		SES at birth	-0.115	0.086	0.185
	Gestational weight gain				
		SES at birth	0.227	0.060	< 0.001
		Maternal age	0.092	0.052	0.074
		Pregestational BMI	-0.083	0.051	0.104
		Smoking during pregnancy	0.067	0.124	0.591
	Type of delivery				
		SES at birth	0.452	0.057	< 0.001
		Maternal age	0.175	0.049	< 0.001
		Gestational weight gain	0.106	0.059	0.071
	Gestational age				
		Maternal age	-0.001	0.047	0.983
		Gestational weight gain	0.127	0.044	0.004
		Type of delivery	-0.091	0.059	0.123
	Birth length					
		SES at birth	-0.039	0.064	0.544
Pregestational BMI			0.170	0.039	< 0.001
		Gestational weight gain	0.117	0.043	0.007
		Type of delivery	-0.098	0.068	0.146
		Gestational age	0.374	0.028	< 0.001
		Sex of the newborn	-0.037	0.043	0.386
Birth weight					
		SES at birth	-0.030	0.047	0.521
		Maternal age	0.091	0.039	0.020
		Pregestational BMI	0.068	0.036	0.061
		Smoking during pregnancy	-0.069	0.070	0.331
		Gestational weight gain	0.090	0.034	0.008
		Type of delivery	0.126	0.047	0.007
		Gestational age	0.135	0.025	< 0.001
		Sex of the newborn	0.014	0.030	0.644
		Birth length	0.655	0.023	< 0.001
	SES of the adolescent				
		SES at birth	0.917	0.060	< 0.001
	“Neither-nor” generation				
		Sex of the newborn	0.158	0.055	0.004
		SES of the adolescent	-0.073	0.070	0.303
	Level of physical activity of the adolescent				
		Sex of the newborn	-0.442	0.037	< 0.001
		Birth weight	-0.072	0.046	0.122
		SES of the adolescent	-0.057	0.053	0.283
		“Neither-nor” generation	-0.114	0.058	0.048
	Alcohol consumption by the adolescent				
		Sex of the newborn	-0.038	0.067	0.570
		SES of the adolescent	-0.001	0.072	0.994
		“Neither-nor” generation	-0.103	0.082	0.207
		Level of physical activity of the adolescent	0.293	0.070	< 0.001

SES: socioeconomic status; BMI: body mass index

The standardized coefficients of the direct effect of the indicator and latent variables on FFM of the adolescent are listed in Table 3 . The total direct and indirect effects including their specific pathways are shown in Table 4 .

**Table 4:** Standardized coefficients, standard errors and p-values of the total, direct and indirect effects of the observed variables and constructs on fat-free mass of adolescents of the RPS cohort of São Luís. São Luís, Brazil, 2016.

Pathways and estimates			Standardized coefficient	Standard error	p
Total, direct and indirect effects					
	SES at birth				
		Total	-0.050	0.045	0.266
	Maternal age				
		Total	-0.053	0.036	0.139
	Pregestational BMI				
		Total	0.152	0.035	< 0.001
		Direct	0.146	0.036	< 0.001
		Indirect	0.006	0.009	0.481
	Smoking during pregnancy				
		Total	0.045	0.057	0.429
	Gestational weight gain				
		Total	0.063	0.041	0.125
	Type of delivery				
		Total	0.092	0.054	0.091
	Gestational age				
		Total	-0.001	0.032	0.972
	Sex of the newborn				
		Total	-0.633	0.024	< 0.001
		Direct	-0.523	0.030	< 0.001
		Indirect	-0.110	0.020	< 0.001
		Indirect specific			
		Via level of physical activity of the adolescent	-0.085	0.021	< 0.001
	Birth length				
		Total	0.026	0.038	0.495
	Birth weight				
		Total	0.106	0.050	0.034
		Direct	0.121	0.051	0.019
		Indirect	-0.015	0.010	0.147
	SES of the adolescent				
		Total	-0.286	0.350	0.413
	“Neither-nor” generation				
		Total	-0.106	0.044	0.015
		Direct	-0.077	0.045	0.084
		Indirect	-0.029	0.015	0.050
		Indirect specific			
		Via level of physical activity of the adolescent	-0.022	0.012	0.075
	Level of physical activity of the adolescent				
		Total	0.207	0.040	< 0.001
		Direct	0.191	0.044	< 0.001
		Indirect	0.016	0.015	0.295
	Alcohol consumption by the adolescent				
		Total	0.054	0.052	0.300

SES: socioeconomic status; BMI: body mass index.

Pregestational BMI had a positive total effect (Standardized Coefficient, SC = 0.152; p < 0.001) and direct effect (SC = 0.146; p < 0.001) on adolescent FFM. The increase of one standard deviation (SD) of pregestational BMI (2.8 kg/m ^2^ ) resulted in an FFM increase of 0.36 kg/m ^2^ during adolescence ( Table 4 ).

Newborn's sex had negative total (SC = −0.633; p < 0.001) and direct (SC = −0.523; p < 0.001) effects, revealing that being a female implied a smaller FFM during adolescence. Newborn's sex also had a negative indirect effect (SC = −0.110; p < 0.001) on FFM, mainly in terms of physical activity of the adolescent (SC = −0.085; p < 0.001). A negative association was found between newborn's sex and level of physical activity (SC = −0.442; p < 0.001) ( Tables 3 and 4 ).

Birth weight had a positive total (SC = 0.106; p = 0.034) and direct (SC = 0.121; p = 0.019) effect on FFM; for each 0.5 kg (1 SD) increase in BW there was a 0.25 kg/m ^2^ increase in FFMI during adolescence ( Table 4 ).

The “neither-nor” generation variable had a negative total effect (SC = −0.106; p = 0.015) on FFM, although without significant direct or indirect effects. The rate of those neither studying nor working represented a reduction in FFM during adolescence ( Table 4 ).

The level of physical activity of the adolescent had a positive total (SC = 0.207; p < 0.001) and direct (SC = 0.191; p < 0.001) effect, with a 0.5 kg/m ^2^ increase in FFM for each 1 SD increase in the level of physical activity ( Table 4 ).

SES at birth and at adolescence had no total effect on adolescent FFM; similarly, the following variables also had no total effect: maternal age, smoking during pregnancy, gestational weight gain, type of delivery, BW, and alcohol consumption during adolescence ( Table 4 ).

Losses to follow up were higher for the poor (78.5%) compared with the better off (73.4%, p = 0.013) and for females (79.8%) compared with males (73.4%, p < 0.001).

## DISCUSSION

In this study, higher values of pregestational BMI and BW and a higher level of physical activity resulted in increased FFM during adolescence. Being a female and not studying or working implied a smaller FFM during adolescence. Regarding the magnitude of the effects, the variables related to birth had a greater effect on adolescent FFM than the variables related to adolescence itself.

Pregestational BMI had a positive effect on FFM, a result also observed in a Brazilian cohort study in which a positive association was detected between pregestational maternal weight and FFMI, FM index and BMI of adolescents ^21^ . This finding might be explained by the fact that increases in maternal weight might be associated with proportional increases in FM and FFM of adolescents. A systematic review of 45 studies concluded that a high pregestational BMI increases the risk of high BW and later overweight/obesity in the offspring ^22^ . However, these studies used BMI as a method to assess nutritional status, so that it was not possible to evaluate in which body compartment an increase occurred ^11^ .

A study conducted in Sweden assessed the body composition of 209 couples and their children by air displacement plethysmography and observed that the FFM of the parents was positively associated with the FFM of the newborn. The magnitude of the effect of parental FFM on newborn FFM was greater for mothers than for fathers, with the authors suggesting that the already known effect of maternal BMI on the BW of the infant was largely due to the effect of maternal FFM rather than FM ^23^ . Strong positive correlations have already been observed between FFM at birth and at four and six years of life ^24^ , showing that this compartment can be preserved along life.

Birth weight had a positive effect on adolescent FFM. The association between higher BW and future overweight/obesity is well known ^25^ , although a large part of the studies used BMI as an indicator of obesity. BMI is correlated with both FM and FFM; thus, the positive lifelong associations already detected between BW and BMI may show the effect of BW on FFM and not on FM ^26^ .

Singhal et al. ^26^ observed that a higher BW was associated with a greater FFM among children and adolescents regardless of sex, age, pubertal status, physical activity, and height. A study published by a consortium which provided cohort data from five low and middle-income countries, including Brazil, also showed that BW was more associated with adult FFM than with FM ^27^ .

The greatest negative effect detected in the analysis was that of females on FFM. There are specific differences in body composition between sexes: women have a relatively larger FM and men a larger FFM ^28^ . These differences are minimal during childhood but become more apparent during adolescence. At the end of this phase and during adulthood, men have on average 1.5 times more FFM than women ^8^ .

The sex of the newborn also showed an indirect effect, being negative via physical activity level. Females were associated with a lower level of physical activity resulting in a smaller FFM during adolescence. A systematic review of 69 published studies show that, in Brazil, the highest prevalence of physical inactivity among adolescents was detected in females, with the magnitude of the difference between sexes ranging from 1% to 29.1% ^29^ . This discrepancy may involve self-efficacy, social support and motivation as factors differently impacting physical activity among women and men ^30^ .

A higher level of physical activity resulted in higher FFM during adolescence. A prospective cohort was formed in Canada to investigate the independent effects of physical activity on FFM, considering the confounding effects of growth and biological maturation. The authors observed that habitual physical activity had an independent influence on the increase in FFM assessed by DEXA during adolescence in both sexes. In addition, they observed that an equal increase in physical activity resulted in a 50% greater FFM accumulation for men than for women ^8^ .

Not studying or working was associated with a smaller FFM during adolescence. The total effect was negative but there was no statistically significant direct or indirect effect, suggesting the sum of effects generated the total effect. Females were was positively associated with not studying or working, and adolescents included in this variable showed lower physical activity levels, a fact that may explain the negative effect of belonging to this group on FFM. There is evidence that spending a period of time not in employment, education or training (NEET) may have a harmful effect on the physical and mental health of young people, with this effect being stronger at younger ages or lasting longer during life ^31^ .

A limitation of our study was the subjects lost to follow-up, especially during the third phase, due to the difficulty in locating the adolescents despite all the search strategies used. Losses to follow-up were higher among females and the poorest adolescents, which may have contributed to underestimating associations in which those strata had higher prevalence. With a larger sample, it may be possible to detect other effects of important determinants.

A strong point is the cohort type of the study, with its advantages regarding reverse causality and the possibility of follow-up of the same population. In addition, equipment considered to be the gold standard and quite accurate for the measurement of each body compartment was used for the evaluation of the body composition of the adolescents. Another relevant point is the statistical method used to analyze the effects of the determinants of FFM during adolescence, i.e., structural equation modeling. This method provides more comprehensive results by estimating various separate and interdependent multiple regression equations, allowing the estimate of total, direct and indirect effects between variables.

The main findings of this study show that the strongest determinant factors for FFM in adolescence are sex, level of physical activity of the adolescent, pregestational BMI, birth weight, and not studying nor working. Females have a greater effect on FFM than all other factors. These findings contribute to the advancement of knowledge in the area, mainly by using a reliable statistical method in a prospective cohort and support the importance of prenatal care of good quality and of a later encouragement of the practice of physical activity, especially among women since this group has a biological tendency to a smaller FFM in later phases of life.
